# Protein Serine/Threonine Phosphatase Type 2C of *Leishmania mexicana*

**DOI:** 10.3389/fcimb.2021.641356

**Published:** 2021-04-15

**Authors:** Alma Reyna Escalona-Montaño, Mariana Zuñiga-Fabián, Nallely Cabrera, Ricardo Mondragón-Flores, Jenny Nancy Gómez-Sandoval, Araceli Rojas-Bernabé, Augusto González-Canto, Laila Gutiérrez-Kobeh, Ruy Pérez-Montfort, Ingeborg Becker, Maria Magdalena Aguirre-García

**Affiliations:** ^1^ Unidad de Investigación UNAM-INC, División de Investigación, Facultad de Medicina, Instituto Nacional de Cardiología Ignacio Chávez., Ciudad de México, Mexico; ^2^ Ciencias Experimentales, Escuela Nacional Colegio de Ciencias y Humanidades Plantel, Naucalpan, Mexico; ^3^ Departamento de Bioquímica y Biología Estructural, Instituto de Fisiología Celular, Universidad Nacional Autónoma de México, Ciudad de México, Mexico; ^4^ Departamento de Bioquímica, Centro de Investigación y Estudios Avanzados (CINVESTAV-IPN), Ciudad de México, Mexico; ^5^ División de Ingeniería en Biotecnología, Universidad Politécnica del Valle de Toluca, Almoloya de Juárez, Mexico; ^6^ Escuela Superior de Medicina, Instituto Politécnico Nacional, Ciudad de México, Mexico; ^7^ Unidad de Investigación en Medicina Experimental, Facultad de Medicina, Universidad Nacional Autónoma de México, Hospital General de México, Ciudad de México, Mexico

**Keywords:** phosphoprotein phosphatase, threonine phosphatases, PP2C, *Leishmania mexicana*, leishmaniasis

## Abstract

Protein phosphorylation and dephosphorylation are increasingly recognized as important processes for regulating multiple physiological mechanisms. Phosphorylation is carried out by protein kinases and dephosphorylation by protein phosphatases. Phosphoprotein phosphatases (PPPs), one of three families of protein serine/threonine phosphatases, have great structural diversity and are involved in regulating many cell functions. PP2C, a type of PPP, is found in *Leishmania*, a dimorphic protozoan parasite and the causal agent of leishmaniasis. The aim of this study was to clone, purify, biochemically characterize and quantify the expression of PP2C in *Leishmania mexicana* (*Lmx*PP2C). Recombinant *Lmx*PP2C dephosphorylated a specific threonine (with optimal activity at pH 8) in the presence of the manganese divalent cation (Mn^+2^). *Lmx*PP2C activity was inhibited by sanguinarine (a specific inhibitor) but was unaffected by protein tyrosine phosphatase inhibitors. Western blot analysis indicated that anti-*Lmx*PP2C antibodies recognized a molecule of 45.2 kDa. Transmission electron microscopy with immunodetection localized *Lmx*PP2C in the flagellar pocket and flagellum of promastigotes but showed poor staining in amastigotes. Interestingly, *Lmx*PP2C belongs to the ortholog group OG6_142542, which contains only protozoa of the family Trypanosomatidae. This suggests a specific function of the enzyme in the flagellar pocket of these microorganisms.

## Introduction

Phosphorylation is the process of adding a phosphate group to a molecule and dephosphorylation the process of removing the same. Addition of phosphates is carried out by protein kinases and removal by protein phosphatases, generally occurring on tyrosine, serine and threonine residues (Cohen, 2002; Wang et al., 2008). There are protein tyrosine phosphatases and protein serine/threonine phosphatases.

The latter type has three families of molecules, one of them being phosphoprotein phosphatases (PPPs), which have great structural and functional diversity. Apart from being responsible for removing the phosphate group from molecules, they regulate a variety of cell functions. The PPP family consists of PP1, PP2A and PP2B phosphatases as well as metal- (Mg^2+^- or Mn^2+^-) dependent protein phosphatases (PPMs). Among PPMs are protein phosphatase type 2C (PP2C) and pyruvate dehydrogenase phosphatase (PDP) (Cohen, 2002; Wang et al., 2008; Virshup and Shenolikar, 2009). Of the 199 phosphatases in the human phosphatome, only 13 are PPMs, but these have a wide range of activity, from regulation of the cell cycle to metabolism and apoptosis (Das et al., 1996; Andreeva and Kutuzov, 2004; Schweighofer et al., 2004; Lammers and Lavi, 2007; Moorhead et al., 2007).

The evidence of PP2Cs in archaea, bacteria, fungi, plants, animals and parasites shows an early evolutionary emergence. Since they evolved from prokaryotes to multicellular eukaryotes, PP2C sequences are very diversified (Fuchs et al., 2013). PP2C enzymes have been cloned and characterized in *Leishmania chagasi* (*Lc*PP2C), *Leishmania major* (*Lm*PP2C) and *Leishmania donovani* (*Ld*PP2C). To our knowledge, however, there is no information in the literature about the function of this enzyme, either in the parasite or cells of the host (Burns et al., 1993; Escalona-Montaño et al., 2017; Jakkula et al., 2018).

The genome of *L. major* was discovered in 2005 (Ivens et al., 2005), and only 2 years later the first analysis of the catalytic domains of protein phosphatases in trypanosomatids was published. A total of 88 protein phosphatases were identified in *L. major*, including 58 serine/threonine phosphatases. Of the latter, 30 are in the PPP family, 13 dephosphorylate the carboxy-terminal domain of RNA polymerase II (F-cell production eukaryotic-like phosphatases), and 15 are PP2C eukaryotic-like phosphatases (Brenchley et al., 2007). The function of these PP2Cs is still unknown.

Our group recently reported the cloning, purification, biochemical characterization and expression level of a protein of *L. major*, concluding that it is a PP2C of threonine/serine protein phosphatases. Additionally, we found PP2C in different *Leishmania* species and described its ultrastructural location in promastigotes, mainly along the flagella (Escalona-Montaño et al., 2017).

*Leishmania* is a dimorphic protozoan parasite and the causal agent of leishmaniasis, a set of neglected tropical diseases (NTDs). According to epidemiological studies, 12 million people are infected worldwide, with 2 million new cases each year (Alvar et al., 2012). Approximately 350 million people are currently at risk of contracting leishmaniasis, mostly in developing countries (Aguirre-Garcia et al., 2018). *Leishmania mexicana*, endemic to the south-eastern region of Mexico, induces various clinical forms of cutaneous leishmaniasis, classified as localized and diffuse leishmaniasis.

Since leishmaniasis is a significant public health problem in México, it is important to determine which enzymes of *L. mexicana* participate in the disease-related signaling pathways. Those showing such activity could possibly be used as a drug target, an immunomodulatory molecule, or a diagnostic marker. The aim of the present study was to clone, purify, biochemically characterize, the PP2C in *L. mexicana*. PP2Cs were also identified in other *Leishmania* species. Based on immunodetection with a transmission electron microscope (TEM), the enzymes were localized in the flagellum and flagellar pocket of promastigotes and amastigotes of *L. mexicana*.

## Materials and Methods

### Culture of *L. mexicana* Promastigotes

Promastigotes of *L*. (V.) *panamensis* MHOM/PA/71/LS94*, L.* (V.) *brazilienzis* MHOM/BR/75/M2903, *L.* (*L.*) *venezuelensis* MHOM/VE/80/PMH3, *L.* (V.) *donovani, L.* (*L.*) *amazonensis* IFLA/BR/67/PH8, and *L.* (*L.*) *mexicana* MNYC/BZ/62/M379 (a generous gift from Dr. Nancy Sarabia, Centro Internacional de Entrenamiento e Investigación Médica in Calí, Colombia (CIDEIM)) were cultivated at 26°C in RPMI 1640 medium containing penicillin (100 units/mL), streptomycin (100μg/mL), and 10% fetal bovine serum. All reagents were purchased from Gibco (Invitrogen Corp, Carlsbad, CA, USA). The cultured promastigotes were washed twice with phosphate-buffered saline (PBS) at pH 7.5 and centrifuged for 10 min at 2,500 × *g* at room temperature (RT).

### Extraction of DNA and Polymerase Chain Reaction Amplification of the Gene for PP2C of *L. mexicana*

Total DNA was extracted from *L. mexicana* promastigotes with TRIZOL. Polymerase chain reaction (PCR) was performed with 1U Taq polymerase (Invitrogen). The optimal magnesium concentration was established by means of testing concentrations in the range of 0-4 mM. Two oligonucleotides were constructed based on alignments of known sequences of PP2C. The sequences for the sense and the antisense oligonucleotides are 5 ′ATGGGSATTCCWCTTCCSA 3 ′ and 5 ′ CCTATTTTCTTGCATTGTTCACT 3 ′, respectively (where S = C/G and W= A/T). Genomic DNA was used as a template under the following conditions: 1 cycle of 5 min at 94°C; 40 cycles of 30 s at 94°C, 1 cycle of 30 s at 51.4°C, 1 cycle of 1 min at 72°C, and 1 cycle of 10 min at 72°C.

### Cloning of the Gene and Sequence Analysis

The amplified gene was ligated to the Topo TA Vector with a cloning kit (Thermo Fisher Scientific) and competent *E. coli* cells (DH5 alpha strain) were transformed. The gene was completely sequenced.

### Expression of the PP2C of *L. mexicana*

The gene was amplified with oligonucleotides containing NdeI and HindIII restriction sites 5’ GGGAATTCCATATGGGCATTCC 3’ and 5 ‘CCCAAGCTTTCACTGCGTCTG 3’, respectively. The released gene was treated with NdeI and Hind III and then ligated to the pET-23b vector (Novagen) expression plasmid, which encodes for proteins that have a carboxyl-terminal His_6_-Tag. The expression of the PP2C of *L. mexicana* was quantified in the Rosetta-gami B (DE3) strain of *E. coli* cells (Novagen), as previously described with some modifications. Expression was induced in bacterial cultures at A 600nm= 0.8 by adding 1.0 mM isopropyl-beta-D-thiogalactoside (IPTG) and culturing the cells for an additional 3 h. Upon completion of this time, the cells were harvested by centrifugation and processed immediately or frozen at -70 to await further use (Escalona-Montaño et al., 2017)

### Purification of Recombinant PP2C of *L. mexicana*

The *Lmx*PP2C protein was purified as reported [11], with some modifications (patent in Mexico, MX/a/2020/011271). Cell cultures were centrifuged in volumes of 100 mL and suspended in 50 mL lysis buffer (50 mM TrisHCl at pH 8, 300 mM NaCl, 1 mM benzamidine, 100µM leupeptin, 2 µg/mL aprotinin and 5 mM imidazole). The suspension was sonicated on a ModelVCX 650 Ultrasonic processor (Ultrasonics, Inc.) five times at 30% amplitude for 60 s intervals, with resting periods of 1 min between each interval. The homogenate was centrifuged at 21,000 × *g* for one hour at 4°C to obtain the supernatant containing the soluble protein. The supernatant was loaded onto an Ni-charged column previously equilibrated with binding buffer (50 mM Tris-HCl at pH 8, 300 mM NaCl and 5 mM imidazole). The recombinant protein (*Lmx*PP2C) was purified and eluted with an elution buffer (50 mM Tris-HCl at pH 8, 300 mM NaCl and 50-500 mM imidazole) and the protein concentration was quantified by the Bradford method (Bradford et al., 1976).

### Production of Polyclonal Antiserum

Anti-*Lmx*PP2C antibodies were generated in rabbits, following the procedure established by Montfort et al. (1994). Briefly, rabbits were injected intramuscularly with 135 µg of recombinant *Lmx*PP2C emulsified in complete Freund’s adjuvant, and the same procedure was repeated 2 weeks later without adjuvant. The immunization was based on two weekly intramuscular injections, after which the animals were bled and antiserum was separated by centrifugation and stored at -20°C. Rabbits were housed at the animal facility of the Research Unit of Experimental Medicine of the Medicine Faculty, UNAM, and handled in accordance with the National Ethical Guidelines for Animal Health NOM-062-ZOO-1999 and the guidelines recommended for animal care by the institutional Ethics in Research Committee.

### Phosphatase Activity Assays

p-NPP substrate. Acid phosphatase activity was determined as described by Dissing et al. (1979). Recombinant *Lmx*PP2C (25 µg) was incubated in the following buffers at 37°C for 60 min: sodium acetate buffer (pH 4.5-5.5), 50 mM MES (pH 6.0 and 6.5), 50 mM HEPES (pH 7.0, 7.5 and 8.0), 50 mM TRIS (pH 8.5-9), or 50 mM CAPS (pH 10-11) added to 10 mM MgCl_2_ and 10 mM *p*-nitrophenyl phosphate [*p*-NPP] in a final volume of 100 μL. Upon completion of this time, the reaction was stopped with 20 μL of 2 N NaOH. Divalent cations (10 mM MgCl_2_ or MnCl_2_) in 50mM HEPES (pH 8) were added and the absorbance was read at 405 nm on a microtiter plate reader, as previously reported with some modifications (Escalona-Montaño et al., 2017).

Phosphopeptides. Tyrosine and serine/threonine phosphatase activity was assessed by employing a non-radioactive tyrosine phosphatase assay system (Promega). The release of inorganic phosphate (P_i_) was monitored by measuring the absorbance of the molybdate-malachite green-phosphate complex. Briefly, 1.2 µg of recombinant *Lmx*PP2C was incubated in a total volume of 100 µL of assay buffer containing 50 mM HEPES at pH 8.0 plus 10 mM MgCl_2._ The reaction was started by adding 50 µM Tyr phosphopeptide-1 substrate [END (pY) INASL] and 50 µM Thr [RRA (pT)VA]. After 30 min at RT, the reaction was stopped with 50 µL molybdate dye/additive mixture. The optical density of the samples was read at 630 nm, using a curve of phosphate density as the standard (Escalona-Montaño et al., 2017).

### Effect of Phosphatase Inhibitors on Recombinant LmxPP2C

The activity of recombinant *Lmx*PP2C (1.2 µg) was evaluated in the presence of specific protein tyrosine phosphatase (PTP) inhibitors: 200 µM sodium orthovanadate, 100 µM sodium tungstate and 50 µM sodium pervanadate. Additionally, serine/threonine phosphatase inhibitors were tested: 5 nM calyculin, 1 µM okadaic acid and 20 µM sanguinarine, the latter a specific inhibitor of PP2C. For the inhibition assays, 100 µL of the reaction mixture was pre-incubated at RT for 15 min before adding the *p-*NPP substrate (all reagents from Sigma-Aldrich, St. Louis, MO, USA). Subsequently, the solution was incubated at 37°C for 60 min and then the reaction was stopped with 20 µL of 2 N NaOH and the absorbance was read at 405 nm on a microtiter plate reader.

### SDS-PAGE and Western Blot

The His-Tag motif was found in recombinant *Lmx*PP2C. The total extract (TE) of bacteria was analyzed by means of sodium dodecyl sulfate-polyacrylamide electrophoresis (SDS-PAGE) in 10% acrylamide gels, and then electrotransferred to nitrocellulose membranes. The blots were incubated with a polyclonal HRP anti-His_6_-Tag antibody at a 1:1000 dilution in TBS-T (200 mM Tris-HCl, 150mM NaCl and 0.005% Tween-20) and washed four times every 10 min with TBS-T. The bands were detected with enhanced chemiluminescent substrate (Super-Signal West Pico Chemiluminescent Substrate, Pierce, Rockford IL, USA), according to the manufacturer’s instructions.

#### Identification of PP2C in Total Extracts of Different Leishmania Species

Promastigotes of *L*. (V.) *panamensis* MHOM/PA/71/LS94*, L.* (V.) *brazilienzis* MHOM/BR/75/M2903, *L.* (*L.*) *venezuelensis* MHOM/VE/80/PMH3, *L.* (V.) *donovani, L.* (*L.*) *amazonensis* IFLA/BR/67/PH8, and *L.* (*L.*) *mexicana* MNYC/BZ/62/M379 were harvested after centrifugation at 2,000 × *g* for 10 min and washing with PBS three times. The pellet containing the parasites was suspended in cold lysis buffer (10 mM imidazole at pH 7.2, 2 μg mL^-1^ leupeptin, 10 μg mL^-1^ aprotinin and 2mM benzamidine) and sonicated to obtain the TE. Ten μg of TE from each *Leishmania* species and 1 µg of *Lmx*PP2C were evaluated with electrophoresis (SDS-PAGE) in 10% acrylamide gels and then electrotransferred onto membranes of low fluorescence. The membranes were blocked with Li-Cor blocking buffer (Lincoln, NE, USA) at RT for 30 min and incubated with antibodies against *Lmx*PP2C using a dilution of 1:1000 in 1% Tween Li-Cor blocking buffer overnight at 4°C. The membranes were washed with PBS-Tween and incubated with a secondary antibody, IRDye 680LT goat anti-rabbit IgG (926-68021, LI-COR Biosciences), at a dilution 1/10000 for 1 h under gentle shaking. The membranes were washed three times with PBS 1X and the proteins were examined on an Odyssey Infrared Imaging system (Li-Cor, Lincoln, NE, USA), according to the manufacturer´s instructions.

### Phylogenetic Analysis of the Ortholog Group of LmxPP2C

The ortholog group of *L. mexicana* PP2C (*Lmx*M.25.0750) was determined with OrthoMCL DB (Li et al., 2003). The phylogenetic tree and branch support of all aligned sequences of PP2C orthologs were constructed with MEGA 6 (multiple sequence alignment) (Tamura et al., 2013). The maximum likelihood method was carried out with an LG model and 5 categories of gamma distribution with invariant sites to find the best-fitting model among those tested. Bootstrap values were based on 100 replicates to estimate support for the nodes of the maximum likelihood tree. The final bootstrapped dendogram was visualized on the Interactive Tree of Life (iTOL) v3 (Letunic and Bork, 2016).

### Multiple Sequence Alignment

*L. mexicana* and *L. major* PP2C sequences (*Lmx*M.25.0750 and *Lm*F.25.0750, respectively) were retrieved from the TriTrypDB database (Aslett et al., 2010). The human sequence NP_066283.1 (PPM1A) was retrieved from the NCBI reference sequence and was used as a seed for multiple sequence analysis. Full-length PP2C protein sequences were aligned on MAFFT v7 (Katoh and Standley, 2013) with L-INS-i (a very slow iterative refinement method recommended for <200 sequences with one conserved domain and long gaps). The final alignment was edited by hand considering the 11 motifs described by Bork et al. (1996), utilizing the alignment explorer of MEGA 6 software (Tamura et al., 2013). Alignment confidence was assessed with the Guidance2 server (Sela et al., 2015).

### Prediction and Structural Alignment of the 3D Model for *Leishmania mexicana* PP2C

The full-length PP2C amino acid sequences from *L. mexicana* and *L. major* were retrieved from the TriTrypDB database with the gene IDs LmxF.25.0750 and LmF.25.0750, respectively. With each sequence, a 3D model was predicted on the I-TASSER server (Zhang, 2008). The final models were selected on I-TASSER, clustering all the decoys on the SPICKER program based on with their pair-wise structure similarity. Up to five models are returned by the program, corresponding to the five largest clusters of structures. The confidence of each model is quantitatively measured by a C-score, calculated in accordance with the significance of threading template alignments and the convergence parameters of the structure assembly simulations. The C-score is typically in the range of [-5, 2], where a C-score of a higher value indicates a model with a greater degree of confidence (Zhang, 2008). The Protein Data Bank (PDB) files of the predicted models were downloaded and the structural alignment to match all structures was performed in the UCSF Chimera program (Pettersen et al., 2004). The crystal structure of human 1a6q was downloaded from the PDB data base.

### Distribution of PP2C in Promastigotes and Amastigotes of *L. mexicana* by Immune Electron Microscopy

The distribution of PP2C in promastigotes and amastigotes of *L. mexicana* was determined by immune electron microscopy (IEM), as previously reported (Escalona-Montaño et al., 2016). Briefly, promastigotes and amastigotes of *L. mexicana* were washed with PBS and fixed with 4% paraformaldehyde and 0.1% glutaraldehyde in PBS at RT for 1 h. Parasites were gradually dehydrated in ethanol, embedded in LR White resin (London Resin, Polysciences, Inc., Warrington, PA, USA), and then left overnight to polymerize in gelatin capsules under UV light at 4°C. Thin sections were cut in an Ultracut E ultra-microtome (Reichert Jung, Austria), mounted on Formvar-covered nickel grids, and incubated with a rabbit antibody against LmxPP2C diluted in PBS-T overnight at 4°C. Grids were washed with PBS-T and incubated with a goat anti-rabbit polyclonal antibody coupled to 10 nm gold particles at RT for 2 h (Zymed, Thermo Scientific, PA, USA). After thorough washings in PBS and distilled water, sections were stained with 2% uranyl acetate and a saturated solution of lead citrate before being viewed on a TEM (JEOL 1400x, JEOL Ltd, Japan). As the negative control, sections were incubated with pre-immune rabbit serum diluted in PBS-T and then with the secondary antibody coupled to gold particles. To recognize the subcellular structures detected by the antibodies, promastigotes and amastigotes were processed in parallel to preserve their ultrastructure, as previously described [28]. Briefly, parasites were fixed in 2.5% glutaraldehyde for 1 h, rinsed with PBS, post-fixed in 1% OsO4 at 4°C, rinsed again, gradually dehydrated in ethanol, and finally embedded in Spurr resin. Thin sections were cut on an Ultracut E ultramicrotome and stained with uranyl acetate and lead citrate. Samples were examined on a TEM at 80 keV. Digital images were taken and processed on Adobe Photoshop software (USA).

### Statistical Analysis

All data is expressed as the mean ± SEM (standard error of the mean). The graph portrays the mean value of three independent assays.

## Results

### Cloning and Purification of PP2C of *L. mexicana*

**Figure 1:**
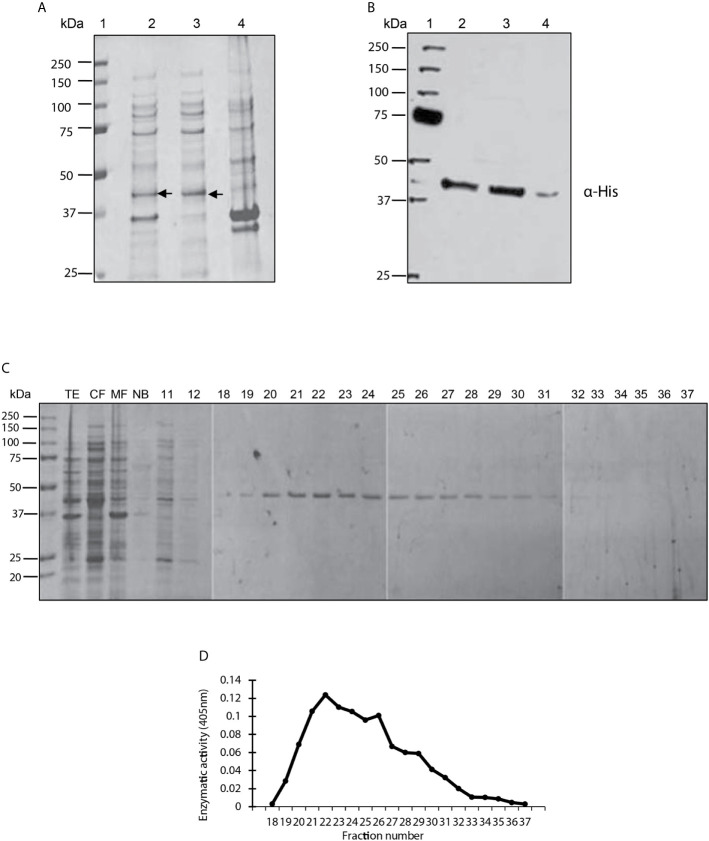
Purification, identification and analysis of enzymatic activity of the PP2C of *Leishmania mexicana*. **(A)** Expression of PP2C of *L. mexicana* in different subcellular fractions of *Escherichia coli* cells. Molecular weight marker (line 1); total extract prepared from the *E.* c*oli* codon plus, induced with IPTG (line 2); cytosolic fraction (line 3); and membrane fraction (line 4). The three extracts were evaluated on SDS-PAGE gel stained with Coomassie blue. **(B)** Western blot analysis of the same sample used in molecular weight marker (line 1); total extract prepared from the *E. coli* codon, induced with IPTG (line 2); cytosolic fraction (line 3); and membrane fraction (line 4). The C-terminal His-Tag was detected with a monoclonal antibody. **(C)** Purification steps, utilizing SDS-PAGE gel stained with Coomassie blue dye. The cytosolic fraction (CF) was applied to a Ni^2+^–NTA resin and the bound material eluted with an imidazole gradient. TE, CF, MF and not bound (NB); 11 and 12 correspond to the washes; 18-37 are the eluents from which the purified protein was obtained. **(D)** The phosphatase activity of the eluents 18-27 was examined by using p-NPP as the substrate.

The amplified *Lmx*PP2C gene was cloned in the pET-23b plasmid to verify that the *E. coli* strain encoded a protein with the characteristics of PP2C phosphatase. The bacterial strain was induced and underwent cell fractionation, from which the TE, cytosolic fraction (CF) and the membrane fraction (MF) were obtained. The three extracts were evaluated on SDS-PAGE gel stained with Coomassie blue (**Figure 1A**). Various proteins of diverse molecular weights can be observed in lanes 2 (TE), 3 (CF) and 4 (MF), including an enriched molecule of 45.2 kDa in lanes 2 and 3 but not in lane 4. Hence, the protein phosphatase was found in the CF.

A Western blot was performed with an anti-histidine antibody to determine whether the recombinant *Lmx*PP2C protein has a sequence of 6 histidines at its extreme carboxyl terminal (**Figure 1B**). The antibody recognized a molecule of approximately 45.2 kDa in the TE (lane 2) and CF (lane 3). A very light phosphorescence is displayed in the MF (lane 4). The results corroborated the presence of histidines in the recombinant protein.

The recombinant protein was purified under nondenaturing conditions with Ni^2+^–NTA resin. Elution was carried out with a linear gradient of imidazole and the eluted fractions were analyzed by electrophoresis (SDS-PAGE), revealing a single protein with an approximate molecular weight of 45.2 kDa (**Figure 1C**). Phosphatase activity was evidenced in fractions 22-25 by a broad peak (**Figure 1D**).

### Identification of PP2C in Different *Leishmania* Species

**Figure 2:**
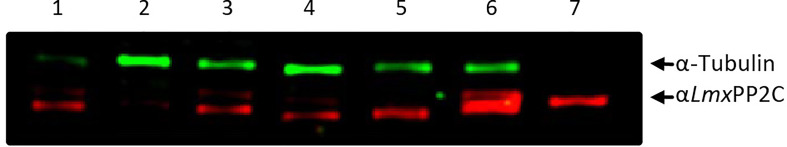
Identification of PP2C in the total extract of promastigotes of different species of *Leishmania*. Immunodetection of the PP2C protein in the total extract (TE) of the promastigotes of *L*. (V.) *panamensis* MHOM/PA/71/LS94 (lane 1)*, L.* (V.) *brazilienzis* MHOM/BR/75/M2903 (lane 2), *L.* (*L.*) *venezuelensis* MHOM/VE/80/PMH3 (lane 3), *L.* (V.) *donovani (lane 4), L.* (*L.*) *amazonensis* IFLA/BR/67/PH8 (lane 5), and *L. (L.*) *mexicana* MNYC/BZ/62/M379 (lane 6). *Lmx*PP2C (lane 7) served as the control and tubulin as a loading control.

The PP2C protein was detected with antibodies against recombinant *Lmx*PP2C (anti-*Lmx*PP2C) applied to TE promastigotes of various *Leishmania* species (**Figure 2**): *L*. (V.) *panamensis* MHOM/PA/71/LS94 (lane 1)*, L.* (V.) *brazilienzis* MHOM/BR/75/M2903 (lane 2), *L.* (*L.*) *venezuelensis* MHOM/VE/80/PMH3 (lane 3), *L.* (V.) *donovani* (lane 4), *L.* (*L.*) *amazonensis* IFLA/BR/67/PH8 (lane 5), *(L.*) *mexicana* MNYC/BZ/62/M379 (lane 6), and *Lmx*PP2C (as the control, lane 7). In all *Leishmania* species (**Figure 2**, lanes 1-6), the protein was of 45.2 kDa, having the same molecular mass as the recombinant protein *Lmx*PP2C (**Figure 2**, lane 7). Tubulin served as the loading control (top panel).

### Biochemical Characterization of PP2C of *Leishmania mexicana*

**Figure 3:**
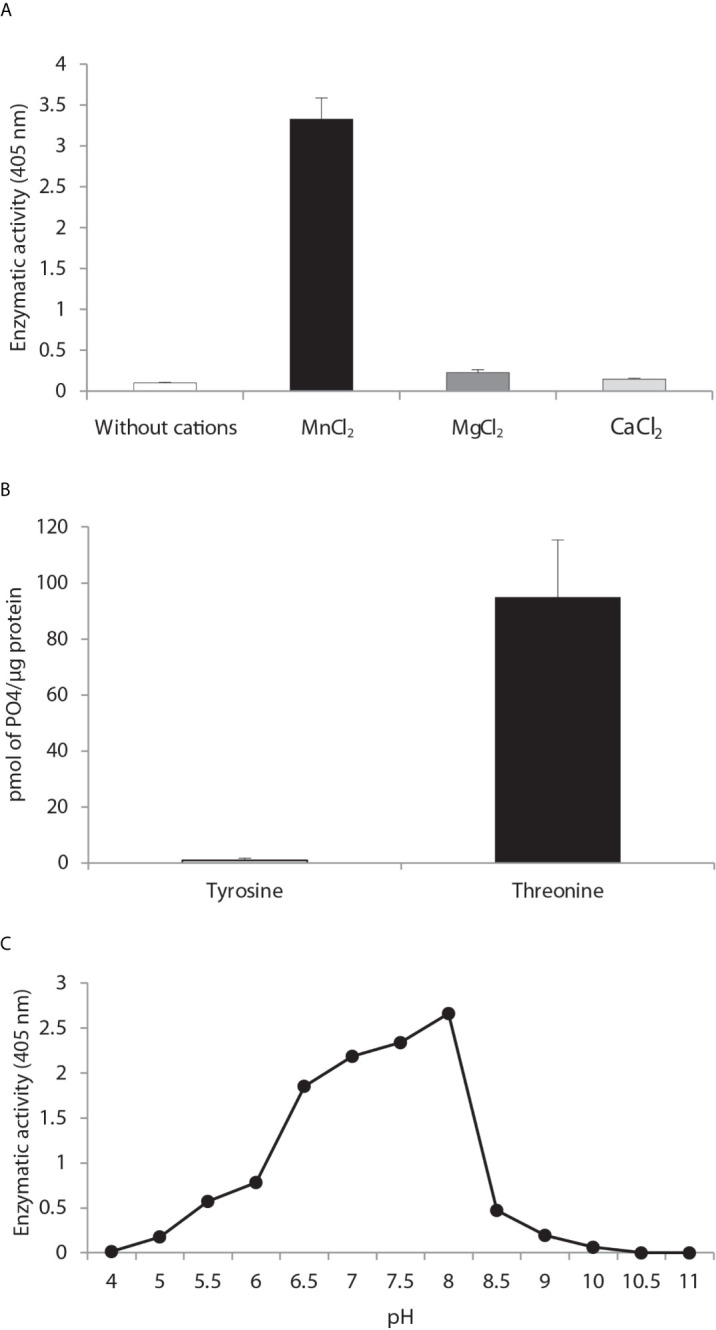
Biochemical characterization of PP2C of *Leishmania mexicana*. **(A)** The effect of MgCl_2_, MnCl_2_ and CaCl_2_ on the activity of PP2C of *L. mexicana* (*Lmx*PP2C) was measured under standard conditions with 10 mM *p*-NPP as the substrate. **(B)** Hydrolysis of [END (pY) INASL] and Thr [RRA (pT) VA] by LmxPP2C. **(C)** Determination of the optimal pH for the activity of *Lmx*PP2C, which was incubated with the appropriate buffers at different pH levels (4–11) in the presence of 10 mM MgCl_2_, with p-NPP as the substrate. Bars depict the mean percentage of relative activity of three independent assays.

*Cation dependence.* Phosphatase activity was measured in the absence of divalent metal ions and in the presence of MnCl_2_, MgCl_2_ and CaCl_2_ (**Figure 3A**) with the generic phosphatase substrate p-NPP. There was a high level of hydrolysis of p-NPP induced by 10 mM of MnCl_2_ and a low level by the divalent cations CaCl_2_ and MgCl_2_. No phosphatase activity was observed in the control sample without cations.

*Substrate specificity.* Substrate specificity was tested by assays with peptides phosphorylated in tyrosine and threonine residues. *Lmx*PP2C dephosphorylated the threonine but not tyrosine substrate (**Figure 3B**).

*Optimal pH.* The pH sensitivity of *Lmx*PP2C was assessed by utilizing *p*-NPP in distinct buffers to determine the optimal conditions for phosphatase activity, which proved to be pH 8 (**Figure 3C**).

### Effect of Some Phosphatase Inhibitors on the Activity of PP2C of *Leishmania mexicana*

The effect of potential inhibitors on *L. mexicana phosphatase* was evaluated on Thr [RRA (pT)VA]. The purified phosphatase was incubated with various inhibitors that target different classes in the serine/threonine protein phosphatase and tyrosine phosphatase families.

**Figure 4:**
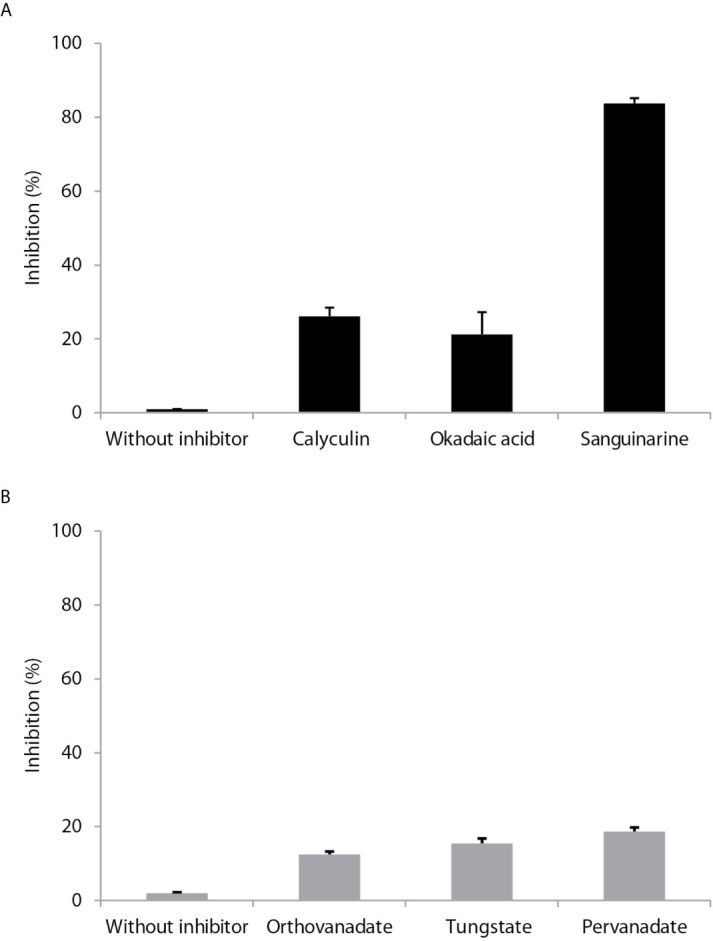
Effect of different inhibitors on PP2C of *Leishmania mexicana*. **(A)** Inhibitors (calyculin and okadaic acid) of serine threonine phosphatases and an inhibitor (sanguinarine) of the PPM family were incubated with PP2C of *L. mexicana* (*Lmx*PP2C), and then phosphatase activity was assessed with *p*-NPP as the substrate. **(B)** Inhibitors of PTPs (orthovanadate, tungstate and pervanadate) were incubated with L*mx*PP2C and then phosphatase activity was evaluated with *p*-NPP as the substrate. Bars represent the mean percentage of relative activity of three independent assays.

Calyculin and okadaic acid, inhibitors of serine/threonine phosphatases, produced 18 and 14% inhibition, respectively, on *Lmx*PP2C. Sanguinarine (a specific inhibitor of PP2C activity) generated an 81% inhibition of *Lmx*PP2C (**Figure 4A**). The phosphatase inhibitors (sodium orthovanadate, tungstate and pervanadate) did not have any significant effect (**Figure 4B**).

### Multiple Sequence Alignment

**Figure 5:**
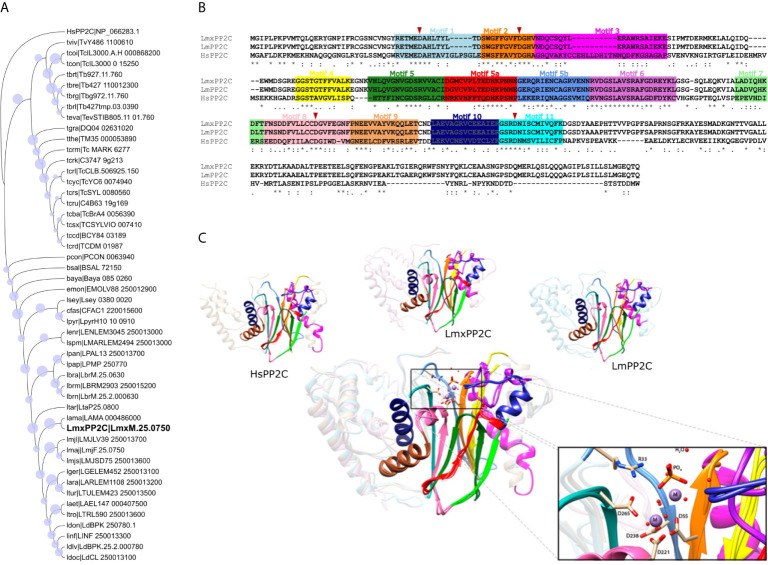
Phylogenetic tree, multiple sequence alignment and structural alignment of *Leishmania* PP2C. **(A)** Analysis of the phylogenetic tree of *Leishmania* PP2C, finding that they belong to the ortholog group OG6_142542 (using the maximum likelihood method based on the LG+G+I model). The analysis involved 50 amino acid sequences. Bootstrap values for each node are shown as a circle. **(B)** Multiple sequence alignment of *L. mexicana, L. major* and human PP2C (*Lmx*M.25.0750, *Lm*F.25.0750 and NP_066283.1, respectively). The 11 conserved motifs are indicated by colored boxes with their corresponding motif numbers in the upper part of the figure. The four aspartates implicated in the catalytic mechanism are conserved in each sequence (red arrowhead). **(C)** Structural alignment of the predicted *Lmx*PP2C, *Lm*PP2C and HsPP2C (1A6Q) carried out on the USCF Chimera program. The 11 conserved motifs of the PP2C catalytic domain are depicted in cartoon representation with distinct colors for each conserved motif. The amino acids participating in the catalysis are portrayed as sticks, while the rest of the protein is illustrated in the background. The structural alignment demonstrates conservation between PP2C sequences.

To examine the conserved sequence motifs for *L. mexicana, L. major* and human PP2Cs, a multiple sequence alignment was performed on the MAFFT program. The colored boxes display the conserved motifs while the red triangles show the conservation of the four aspartate residues that are crucial in the catalytic activity. *Lmx*PP2C has a 98.77% sequence similarity with *Lm*PP2C and 44.10% with human PP2C, indicating a different role of these enzymes in *Leishmania* spp. versus humans (**Figure 5A**).

### Phylogenetic Tree Analysis of the Ortholog Group of *Leishmania* PP2C

*Lmx*PP2C belongs to the ortholog group OG6_142542, which contains 49 species belonging to the family Trypanosomatidae. To investigate the phylogenetic relationship of all PP2C protein sequences in this ortholog group with human PP2C (NP_066283.1, OG6_100270), a phylogenetic tree was constructed based on the alignments of PP2C domains using the maximum likelihood method. According to the phylogenetic analysis, these 49 PP2C sequences are grouped by taxa. Since human PP2C is found in a distinct ortholog group, it probably has a different function than that of the enzymes of *Leishmania and other Trypanosomatidae* species (**Figure 5B**).

### Prediction and Structural Alignment of the 3D Model of PP2C of *Leishmania mexicana*

To identify structural similarities and differences between *Lmx*PP2C, *Lm*PP2C and human PP2C, firstly a homology prediction was made using the I-TASSER server and then the structural alignment was visualized in the UCSF-Chimera program. The eleven motifs of PP2C in the three species are shown in colors, as was done in the multiple sequence alignment. The catalytic PP2C domain is the typical β-sheet sandwich surrounded by α-helices. In the case of *L. mexicana*, the catalytic site of PP2C is localized in a cleft between two central β sheets formed by aspartate residues D38, D55, D221 and D265 as well as arginine R33*. *The structure alignment of these three species indicates that they are homologous, containing conserved elements of the PP2C family with a few changes in the motif 3 secondary structure and the C-terminal length (**Figure 5C**).

### Ultrastructural Localization of PP2C in Promastigotes and Amastigotes of *L. mexicana*

#### Distribution of PP2C in Promastigotes and Amastigotes of *L. mexicana*

**Figure 6:**
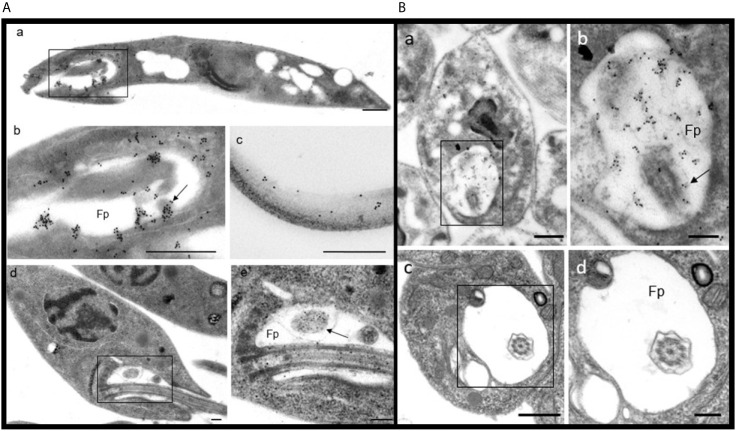
Distribution of PP2C in promastigotes and amastigotes of *L. mexicana*. **(A)** Immune electron microscopy of LmxPP2C in promastigotes. The micrographs in A are of low (Images a and d) and high magnification (images b, c and e) of promastigotes. Image (b) corresponds to a magnification of the inset in (a). Micrographs (d) and (e) correspond to the control for the ultrastructure of a promastigote. The image (e) represents a magnification of the inset in (a). Scale bars = 500 nm. **(B)** Immune electron microscopy of LmxPP2C in amastigotes. The micrographs are of low (images a and c) and high magnification (b and d) of amastigotes. Image (b) portrays a magnification of the inset in (a). Images (c) and (d) correspond to the control for ultrastructure of an amastigote. Fp, flagellar pocket. Scale bars in a and c = 500 nm; scale bars in b and d = 200 nm.

PP2C was observed in promastigotes of *L. mexicana* by IEM (based on an accumulation of the gold label), localized in the flagellar pocket and particularly in the corresponding vesicles (**Figures 6A**(**a**, **b**), arrow). The label was also detected along the flagellum (**Figure 6A**, image c). In order to recognize the precise location of the labeling with gold particles, ultrastructural micrographs of promastigotes and of the flagellar pocket are herein included (**Figure 6A**, Images d and e). Spheroid structures in the flagellar pocket were labeled by the antibody against LmxPP2C and are easily seen in the micrograph of the respective control of ultrastructure as electron-dense vesicles (**Figure 6A**, images b and e respectively, arrow). Samples incubated with pre-immune serum displayed no IEM labelling of the parasites (data not shown).

The distribution of PP2C in amastigotes of *L. mexicana* was viewed by IEM, finding a specific location of the immune gold label in the flagellar pocket (**Figure 6B**, images a and b). The flagellum located in the flagellar pocket (Image b, arrow) was poorly marked with gold particles. In contrast, the electron-dense vesicles and the fuzzy material located in the flagellar pocket were heavily marked by the immune gold label (Image b). There was scarce labeling in the cytoplasm (data not shown). The precise location of the gold labelling was determined based on the amplified images of the respective control of ultrastructure of amastigotes and the flagellar pocket (**Figure 6B**, Images c and d). Thin sections incubated with pre-immune serum did not exhibit any labelling of the parasites (data not shown).

## Discussion

Protein phosphatases have served as a target for the design of anti-cancer drugs (Le Guezennec and Bulavin, 2010). The PP2C delta phosphatase (Wip) plays an important role in homeostasis as well as in the pathogenesis of several human diseases. Wip1 is considered a negative regulator of the p53 oncogene pathway in tumor lines (Goloudina et al., 2016). PP2Cs are monomeric enzymes that require metal cations (Mg^+2^ or Mn^+2^) for their enzymatic activity. They are comprised of a catalytic domain with 11 conserved motifs containing aspartic acid. Their catalytic activity is highly regulated and may have enormous therapeutic potential (Baskaran and Velmurugan, 2018).

In parasites, protein phosphatases of serine/threonine and tyrosine have been cloned and characterized. Our group has characterized protein phosphatases in protozoan parasites such as *L. major* and *Cryptosporidium parvum* (Escalona-Montaño et al., 2017; Gómez-Sandoval et al., 2020). In the current contribution, the recombinant protein PP2C of *L. mexicana* was cloned and purified.

PP2C protein phosphatases have been characterized in *Leishmania*, including the PP2C of *L. chagasi* (*Lc*PP2C), *L. major* (*Lm*PP2C) and *L. donovani* (*Ld*PP2C), (Burns et al., 1993; Escalona-Montaño et al., 2017; Jakkula et al., 2018). These enzymes share very similar biochemical characteristics. For example, purified *Ld*PP2C is enzymatically active, and according to an inhibition study, sanguinarine likely acts as a non-competitive inhibitor (at pH 3.5-8.5). The molecular weight of the purified protein was 45 kDa (Jakkula et al., 2018). *Lc*PP2C was reported to have a molecular weight of 42 kDa in both the infective promastigotes and the tissue amastigotes of *L. chagasi* and *L. amazonensis*.

To verify the relationship between *Lc*PP2C and mammalian PP2Cs observed in predicted amino acid sequence comparisons, enzymatically active *Lc*PP2C was expressed. Purified *Lc*PP2C readily dephosphorylated [32P] casein in an Mg^+2^-dependent and okadaic acid-independent manner (Burns et al., 1993). In *L. major*, there was a protein of 44.9 kDa with PP2C activity, the latter being dependent on divalent cations (Mg^+2^ and Mn^+2^) and found to be optimal at a pH of 8.5 when using phosphothreonine as the substrate. Sanguinarine inhibited the activity of recombinant *Lm*PP2C (Escalona-Montaño et al., 2017).

The present results reveal that recombinant PP2C of *L. mexicana* has a molecular weight of 45.2 kDa, is Mn^+2^-dependent, and has optimal activity at pH 8 with p-NPP as the substrate. It preferentially dephosphorylated threonine (versus tyrosine), an activity inhibited by sanguinarine. The Mn^+2^-dependency has been described for other microorganisms, such as a *Chlamydia* spp. (Claywell et al., 2018).

The similarity of PP2C between distinct species of *Leishmania* makes this enzyme an important subject of research for the development of diagnostic tools, vaccine immunogens, and drugs for treating leishmaniasis. However, the function of PP2C enzymes in the *Leishmania* species is still unknown. *Ld*PP2C affects the host innate immune response by upregulating pro-inflammatory cytokines (TNF-α and IL-6) as well as nitric oxide (Jakkula et al., 2018), while *Lc*PP2C elicits a strong proliferative response of T cells in patients with leishmaniasis (Burns et al., 1993).

In both the promastigote and amastigote forms, there is an invagination of the plasma membrane at the base of the flagellum (the flagellar pocket). This key feature of trypanosomatid cells is central to several vital processes, including differentiation, endo/exocytosis, flagellum assembly, and the definition of surface membrane boundaries. Such processes are critical for the cell biology underlying the life cycle of *Leishmania* (Sunter et al., 2018). The location of PP2C in the flagellum of *L. major* suggests a potential regulatory activity in this organelle. Nevertheless, the functional role of *Lm*PP2C has yet to be established (Escalona-Montaño et al., 2017). In other trypanosomatid parasites, such as *Trypanosoma rangeli*, a PTP was associated with the parasite flagellum, although its function is unknown (Prestes et al., 2012). In most of these parasites, the PP2C enzymes are mainly located in the cytosol. During invasion, the *T. gondii* parasite secretes PP2C from the rhoptries to deliver it to the host cell and target the respective nucleus (Gilbert et al., 2007).

In *L. mexicana*, the presence of *Lmx*PP2C in the flagellar pocket of the parasite indicates that the enzyme is probably discharged from Golgi-derived multivesicular bodies in the same location, which would explain why the labelling was found in the vesicles of promastigotes and amastigotes. Hence, phosphorylation is likely carried out by yet unidentified kinases in the Golgi apparatus involved in a reciprocal regulatory relationship with *Lmx*PP2C.

Since PP2C is located in the flagellar pocket of promastigotes (an important organelle in *Leishmania*), it represents a possible drug target or diagnostic marker of leishmaniasis. Comparative analysis of gene expression across promastigote and amastigote forms revealed a total of 3,832 genes differentially expressed between promastigotes and intracellular amastigotes. During differentiation to amastigotes, a large proportion of the downregulated genes are linked to the function of the motile flagellum, while the upregulated genes encode cell surface proteins, transporters and peptidases, as well as many other proteins yet to be defined (the undefined genes comprise 293 of the 936 novel genes) (Fiebig et al., 2015). The *Lmx*M.25.0750 gene has higher expression in promastigotes than amastigotes, which is correlated with its location in the flagellar pocket. Given that *Lmx*PP2C belongs to the ortholog group OG6_142542 (containing only protozoa of the Trypanosomatidae family), it probably has a specific role in the flagellar pocket of these microorganisms.

The finding of a recombinant *Lmx*PP2C protein opens a new field of research on its use as a drug target, an immunomodulatory molecule, and/or a diagnostic marker for leishmaniasis. Further research is needed on the function of this protein in the parasite, since it may be a key molecule in *L. mexicana* and in the pathogenesis of leishmaniasis.

## Data Availability Statement

The raw data supporting the conclusions of this article will be made available by the authors, without undue reservation.

## Ethics Statement

The protocol for the experiments carried out with animals in this study was reviewed and approved by the Ethics in Research Committee and the Internal Committee on the Care and Use of Lab Animals (CICUAL, according to the name of the committee in Spanish), both of the Medicine Faculty of the Universidad Nacional Autónoma de México. The fundamental purpose of these committees is to evaluate and if appropriate authorize the use of protocols for the care and use of lab animals in research or teaching, with the aim of regulating such practices so that they conform to the Mexican norms established in the law known as NOM-062-ZOO-1999.

## Author Contributions

The purification of *Lmx*PP2C was performed by AE-M. The experimental characterization of PP2C activity and the Western blot assay were carried out by MZ-F. The characterization of *Lmx*PP2C was supervised and validated by IB in her lab. The gene cloning experiments were designed by RP-M, who supervised and validated them in her lab. The gene encoding *Lmx*PP2C was cloned and overexpressed by NC. *Lmx*PP2C was localized in promastigotes and amastigotes with a transmission electron microscope (TEM) by RM-F. The parasites used for localizing LmxPP2C with a TEM were provided by IB. The in silico model of PP2C was constructed by JG-S. The polyclonal antibodies against *Lmx*PP2C were generated by AG-C. The statistical analysis and the creation of the figures was done by AR-B. The analysis of the results involved all authors under the direction of IB and MA-G. The conceptualization of the study and funding acquisition for the experimental work was the responsibility of MA-G, and the manuscript was structured by MA-G. The original draft was prepared by AE-M and MA-G, and the writing was reviewed and edited by AE-M, JG-S, LG-K and MA-G. All authors contributed to the article and approved the submitted version.

## Funding

This research was funded by the Secretaría de Educación Pública - Consejo Nacional de Ciencia y Tecnología (SEP-CONACyT, Mexico), grant number 284018, and partially sponsored by the DGAPA-PAPIIT, grant number IN218619, given to MA-G.

## Conflict of Interest

The procedure presently used to purify and quantify the expression of PP2C in *L. mexicana* has been patented in Mexico (MX/a/2020/011271) by AE-M and MA-G.

The remaining authors declare that the research was conducted in the absence of any commercial or financial relationships that could be construed as a potential conflict of interest.
